# PARP inhibitors in non-ovarian gynecologic cancers

**DOI:** 10.1177/17588359241255174

**Published:** 2024-06-12

**Authors:** Italo Fernandes, Rania Chehade, Helen MacKay

**Affiliations:** Sunnybrook Odette Cancer Centre, Toronto, ON, Canada; Sunnybrook Odette Cancer Centre, Toronto, ON, Canada; Sunnybrook Odette Cancer Centre, Sunnybrook Research Institute, 2075 Bayview Avenue, Toronto, ON M4N 3M5, Canada

**Keywords:** clinical trials, homologous recombination deficiency, novel therapies, PARP inhibitor, predictive biomarker

## Abstract

Poly(ADP-ribose) polymerase (PARP) inhibitors (PARPis) have transformed the treatment of ovarian cancer, particularly benefiting patients whose tumors harbor genomic events that result in impaired homologous recombination (HR) repair. The use of PARPi over recent years has expanded to include subpopulations of patients with breast, pancreatic, and prostate cancers. Their potential to benefit patients with non-ovarian gynecologic cancers is being recognized. This review examines the underlying biological rationale for exploring PARPi in non-ovarian gynecologic cancers. We consider the clinical data and place this in the context of the current treatment landscape. We review the development of PARPi strategies for treating patients with endometrial, cervical, uterine leiomyosarcoma, and vulvar cancers. Furthermore, we discuss future directions and the importance of understanding HR deficiency in the context of each cancer type.

## Plain language summary

PARP inhibitors in non-ovarian gynecologic cancers

Poly(ADP-ribose) polymerase (PARP) inhibitors (PARPi) have transformed the way ovarian cancer is treated, especially for patients whose tumors have specific genetic issues affecting their ability to repair DNA. Over time, PARPi are being used for certain groups of patients with breast, pancreatic, and prostate cancers. More recently, their potential to help people with other types of gynecologic cancers than ovarian have been studied. In this review, we explore the reasons behind looking into PARPi for these non-ovarian gynecologic cancers. We analyze the clinical data and compare it to the current treatment options available, focusing on endometrial, cervical, uterine leiomyosarcoma, and vulvar cancers. Additionally, we discuss about future directions and stress the importance of understanding the specific DNA repair context for each type of cancer. Especially, we discuss the tests that aims to define who may benefit from the drug, with focus on the homologous recombination deficiency.

## Introduction

Poly(ADP-ribose) polymerase (PARP) inhibitors (PARPis) are established as part of the standard of care management for subsets of patients diagnosed with epithelial ovarian cancer, specifically in the maintenance setting following initial platinum-based chemotherapy.^1^ Beyond ovarian cancer, they have been investigated in a number of different tumor types including breast,^2^ pancreatic,^3^ and prostate^4^ cancers. PARPi are particularly active in cancers which have an impaired ability to repair double-strand DNA breaks (DSBs) *via* the homologous recombination (HR) pathway.^5^ HR deficiency (HRD) occurs as a result of a number of mechanisms most notably through pathogenic mutations, or epigenetic modification, of *BRCA1/2*.^6,7^ Although their clinical efficacy is well-established in ovarian cancer, emerging data suggest a potential therapeutic role for PARPi in other gynecologic cancers. This review aims to assess the current evidence for the use of PARPi in non-ovarian gynecological cancers, discussing the rationale, ongoing trials, and outlining future perspectives.

## Background

### DNA repair, synthetic lethality, and PARPi

**Figure 1.**
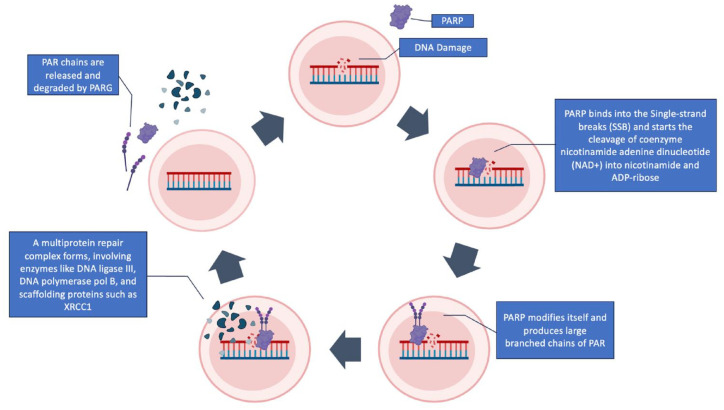
PARP and DNA damage response. PARP-1 detects SSBs and transduces signals, initiating the synthesis of negatively charged PAR polymers on target proteins through binding to adjacent DNA. This process, known as PARylation, involves the cleavage of coenzyme NAD+ into nicotinamide and ADP-ribose. Consequently, large PAR polymers are produced, forming a multiprotein repair complex that includes enzymes like DNA ligase III, DNA polymerase pol B, and scaffolding proteins such as XRCC1. After ADP-ribosylation, PARP-1’s DNA affinity decreases, leading to its release. The repair PAR polymers undergo degradation facilitated by PARG. NAD, nicotinamide adenine dinucleotide; PAR, poly(ADP-ribose); PARG, poly(ADP-ribose) glycohydrolase; PARP-1, poly(ADP-ribose) polymerase-1; SSBs, single-strand breaks; XRCC1, X-ray repair cross-complementing 1.

Cells undergo DNA damage in response to various stressors. The repair of DNA damage, essential to genomic stability and cell survival, is mediated primarily through two main pathways: homologous recombination repair (HRR) of DSB and base excision repair (BER) of single-strand breaks (SSBs).^8^ PARP is a large family of 18 proteins involved in various cellular processes, with a notable role in DNA repair.^9^ Their primary function is to repair SSBs in DNA *via* the BER pathway.^9,10^ This is described in Figure 1. Inhibiting PARP-1 leads to a failure in the repair of SSBs, causing the formation of a DSB which can, in normal cells, be repaired by the high-fidelity HR pathway.^11,12^ Cells which have lost HRR^13,14^ become more reliant on other DNA repair pathways with lower fidelity such as nucleotide excision repair (NER) and the non-homologous end joining (NHEJ) pathway.^15^ This reliance on lower fidelity repair pathways results in lethal levels of DNA damage and forms the rationale for PARPi use.

Olaparib was the first PARPi approved by the United States Food and Drug Administration for use in the clinic. This, and other first-generation PARPi, primarily target PARP1 and PARP2 inhibiting catalytic activation with half maximal inhibitory concentrations (IC50) in the nanomolar range.^16,17^ Agents vary in their selectivity for different members of the PARP family, for example, veliparib demonstrates over 100-fold higher selectivity for PARP1/2 compared to olaparib which exhibits a 15-fold selectivity. In addition to targeting catalytic activity, PARPi can trap PARP1 (and 2) on DNA and impair recruitment of BRCA1, activation of NHEJ, and cause destabilization of stalled replication forks.^18–20^ Some first-generation PARPi inhibit other non-PARP targets. Niraparib, for instance, inhibits deoxycytidine kinase in addition to PARP.^21^ This variability in selectivity potentially contributes to both the efficacy and toxicity of the agents. To date, clinical trials directly comparing different PARPi have not been performed. It remains unknown whether different tumor, or molecular, subtypes require different PARP inhibitory strategies to achieve optimal efficacy. Next-generation PARPi are more selective for PARP1. AZD5305 induces PARP1 trapping while sparing PARP2.^22,23^ Targeting PARP1 alone has the potential to widen the therapeutic window and may allow PARPi combinations with other agents or modalities, such as chemotherapy, which to date have been challenging due to myelosuppression.^23^

### Measuring HRD

Inherent in the original pre-clinical development of PARPi was the requirement for *BRCA1/2* pathogenic variants in cells in order for ‘Synthetic lethality’ to occur.^24^ Subsequently, it became apparent that potential biomarkers of response to PARPi (and indeed platinum-based chemotherapy) extend beyond *BRCA1/2* mutations. Thus, other mechanisms have been proposed as potential predictors of PARPi sensitivity, as alternate mechanisms of functional HRR loss, and silencing mutations in additional genes involved in HRR, such as *ATM*, *NBS1*, *RAD51*, *FANC*, *ATR*, *BAP1*, *PALB2*, *FANCA*, *FANCC*, *FANCE*, *FANCF*, *CHEK1*, *BLM*, *BRIP1*, *CHEK2*, *RAD51C*, *RAD51D*, *RAD51B*, and *CDK1.* Furthermore, in ovarian cancer, we have learnt that biomarkers of response or, perhaps more importantly, resistance are not static and may evolve over time.^25^ Assays developed in ovarian cancer to identify patient populations who might benefit from PARPi include sequencing of HR genes, promoter methylation assays, genomic scar detection based on copy number changes, mutational signature, transcriptional signatures, and functional assays.^26^

The majority of assays used in clinical trials (and clinic) combine genomic profiling with a genomic instability/scarring score in order to predict the presence of an HRD phenotype. Genomic signatures of chromosomal instability include patterns of genomic loss of heterozygosity (gLOH), number of telomeric imbalances, and large-scale transitions or chromosomal changes such as translocations or deletions. Functional assays, such as detection of RAD51 foci following exposure to platinum or ionizing radiation, have also been reported. Even for ovarian cancer, optimal HRD testing remains controversial. There is only partial agreement on which parameters are required to determine a predictive HRD phenotype, and assays use different ‘cut off points’ even across trials.^26^ There is a need to better understand the discordance between assays assessing HR status. In expanding PARPi therapeutic strategies into the non-ovarian gynecologic cancer patient populations, it is essential that we understand whether measures of both the ‘cause and consequence’ of HRD are necessary to determine HR status for each tumor type. This will require pre-clinical and clinical research to understand the effect of HRR mutations, beyond *BRCA1/2*, in the context of each type of cancer as the impact may differ. It is important that the methodology is fully described in any study where HR status is included. Collaboration between researchers to understand how we optimize predictive biomarker testing will be essential if we are to optimize PARPi use, particularly in rare cancers.

Great interest is currently focused on identifying both primary and acquired biomarkers of resistance to PARPi in ovarian cancer. Studies involving liquid biopsies and circulating tumor DNA to understand the incidence of reversion mutations have shown particular promise.^27^ Inclusion of appropriate tissue/blood collections as part of clinical trials in non-ovarian gynecologic cancer is desirable to identify biomarkers of PARPi sensitivity and resistance. Once again collaboration and clear description of the methodology employed will be important if we are to see biomarker testing evolve.

### PARPi in the clinic

PARPi in the clinic are generally well-tolerated oral agents. The main adverse events (AEs) are nausea, fatigue, and thrombocytopenia. Longer-term use of PARPi has been associated with the development of Myelodysplastic Syndrome (MDS) and acute myeloid leukemia (AML), with a World Health Organization Pharmacovigilance database demonstrating significantly increased risk of 2.63 [95% confidence interval (CI), 1.13–6.14, *p* = 0.026].^28^ It is, therefore, essential to have long-term follow-up on all patients receiving PARPi, including non-ovarian gynecologic cancer patients, so that the risk for individual patient populations can be defined. As the field evolves, careful consideration of duration of PARPi treatment will also be required if we are to minimize risks and optimize any benefits.

## Endometrial cancer

Endometrial cancer (EC) is the most common gynecologic malignancy, with approximately 417,317 new cases worldwide each year.^29^ The incidence of EC is increasing largely attributed to the rise in obesity, increased life expectancy, and changes to gynecologic practice and childbearing.^30^ Recent risk stratification models for EC have evolved to include molecular classification. In 2013, the Cancer Genome Atlas analysis identified four distinct, prognostic, molecular subgroups. Subsequently, pared down classifiers were developed and validated to enable identification of these subgroups in the clinic: P53abn and mismatch repair deficient (dMMR) using immunohistochemistry (IHC); limited sequencing of the exonuclease domain of DNA polymerase epsilon (POLE) to identify POLE-mutated tumors, and the remainder labeled nonspecific molecular subtype (NSMP).^31,32^ How these potentially relate to HRD in EC will be discussed below and is an active area of research.^33^

### Rationale for exploring PARPi in EC

The presence of germline pathogenic variations in HRR genes has been well documented in EC. Among 11 studies (*N* = 1613 patients, *N* = 1129 serous histology), the prevalence of g*BRCA1/2* pathologic variants was 4.3%, predominantly g*BRCA1* (71.4%). The risk of developing EC in patients with known g*BRCA1/2* mutations varies across studies, with some reporting an increased risk for both g*BRCA 1* and *2*, whereas others suggesting an increased risk limited to g*BRCA1* mutations.^34^ In addition, pathogenic germline mutations have been reported in other HRR genes including *BRIP1* and *RAD51* in EC patients.

Somatic mutations in HRR genes that could lead to an HRD phenotype have been observed in EC. Next-generation sequencing (NGS) of tumors from several cancer sites, including 1475 EC, identified potentially pathogenic mutations in 34.4% of EC cases. The most common genes included: *ARID1A* (27%), *ATM* (4.61%), *ATRX* (3.13%), and *BRCA2* (3.05%).^35^ However, as discussed above, a greater understanding of what role these mutations play in the development of HRD (and PARPi response), if any, in EC is required.

Assays developed in ovarian cancer to measure HRD have been investigated in EC. Siedel *et al.*^36^ examined 253 endometrioid EC samples using the Myriad myChoice assay, demonstrating that a higher HRD score (cut off score ⩾4) was associated with worse disease-free survival.^36^ de Jonge *et al.*^37^ conducted a functional HR study on fresh tumor samples (*N* = 36) assessing the ability of replicating tumor cells to accumulate RAD51 foci induced by ionizing radiation. The investigators concluded that evidence of HRD was limited to non-endometrioid histology, occurring in 46% of cases.^37^ Genomic instability scores have been assessed in EC using various cut offs. In one study defining gLOH high as >16%, 24% of copy number high (P53Abn) EC exhibited high gLOH compared to 3% across the other molecular subgroups.^38^ This was also observed in the correlative analysis from the clinical trial NRG-GY012, where gLOH high status (⩾11%) occurred in 35.6% of cases and was only observed in *TP53* mutated cancers.^39^

Early pre-clinical studies with olaparib support investigation of PARPi in EC. Clonogenic assays revealed variable sensitivity across cells lines, with SF50 (concentration to inhibit cell survival to 50%) values of 100 nM or less in 4 (25%) of cell lines.^40^ Hansen *et al.*^41^ reported on mouse models bearing EC cell lines treated with olaparib. There was a significant reduction in tumor weight following PARPi administration. Reduced proliferation and increased apoptosis were observed in cell lines regardless of HRD score, although greater response was noted in those with higher HRD scores.^41^

Loss of phosphatase and tensin homolog (PTEN) occurs in up to 78% of endometrioid EC cases.^31^ The role of PTEN in the HR pathway is controversial, although it has been reported to regulate *RAD51* expression.^42^ Initial reports from Dedes *et al.*^43^ suggested, in pre-clinical models, that *PTEN* null EC cell lines displayed increased sensitivity to a PARPi compared to those that expressed wild-type *PTEN*.^43^ However, subsequent studies could not corroborate this finding, as Miyasaka *et al.*^40^ found no correlation with olaparib sensitivity and PTEN loss across 16 EC cell lines. It remains unclear in the clinic whether loss of PTEN is associated with PARPi sensitivity.

ARID1A mutation occurs in approximately 46% of endometrioid EC, with loss of expression reported in 26% of cases.^44^ Loss of ARID1A impacts cell cycle control and DNA repair. ARID1A, *via* its interaction with ATR and Rad3, is recruited to DSB facilitating DNA processing and producing SSB.^45^ Shen *et al.*^46^ investigated olaparib, veliparib, and rucaparib in ARID1A knocked down breast and colon cells lines and in animal models. They demonstrated potential efficacy for PARPi alone or in combination with ATR inhibitors.

Taken together, both the tumor based and pre-clinical EC data strongly support investigation of PARPi in the clinic.

### Current treatment for EC

Given that early-stage EC is typically cured by surgery, with or without adjuvant radiotherapy, the potential role for PARPi in the management of EC lies in treating recurrent/advanced cancers or those with high-risk features. The incorporation of molecular subgroups is changing how we approach treatment and how we interpret (and design) clinical trials. Initial studies of PARPi in EC were conducted in ‘unselected’ patient populations. However, as data have accumulated PARPi studies focusing on, or enriched for, the P53abn subgroup have gained attention.

The backbone of systemic treatment for advanced EC has long been platinum-based chemotherapy.^47^ However, response rates to platinum in EC^47^ are lower and less durable than those seen in high grade serous ovarian carcinomas.^45^ This clinical observation alone suggests that the HRD phenotype may differ between EC and ovarian cancers.^48^ Second-line treatment of EC, until recently, consisted of chemotherapy or immunotherapy with or without a receptor tyrosine kinase inhibitor (RTKi) based on the presence or absence of MMR proteins.^49^ Beyond first-line treatment options are limited. Hence, understanding if there is a subset of patients who might benefit from PARPi alone or in combination is of high interest.

In 2023, two clinical trials established a new standard of care for EC. They investigated immunotherapy [programmed death 1 (PD1) inhibitor]delivered concurrently with chemotherapy followed by maintenance in the first-line treatment of recurrent or metastatic EC. NRG GY018 had co-primary endpoints of progression-free survival (PFS) in the dMMR and pMMR (MMR proficient) EC patient populations. In the dMMR cohort, median PFS at 12 months was 74% in patients receiving pembrolizumab compared to 38% in the patients who received placebo, hazard ratio (HR) 0.30; 95% CI, 0.19–0.48; *p* < 0.001. In the pMMR cohort, median PFS was 13.1 months with pembrolizumab *versus* 8.7 months with placebo, HR 0.54; 95% CI, 0.41–0.71; *p* < 0.001.^50^ In the RUBY study, for all evaluable participants, PFS rate at 24 months was 36.1% (95% CI, 29.3–42.9) in the dostarlimab arm and 18.1% (95% CI, 13.0–23.9) for the placebo arm, HR 0.64, 95% CI, 0.51–0.80; *p* < 0.001. In a pre-planned exploratory analysis, dostarlimab demonstrated a significant improvement in PFS in patients with dMMR EC compared to placebo, HR 0.28, 95% CI, 0.16–0.50; *p* < 0.001.^51^ Based on the results of NRG GY018 and the dMMR analysis of the RUBY trial, carboplatin/paclitaxel combined with a PD1 inhibitor became the standard of care for dMMR EC patients. The role of first-line combinations with a PD1/programmed death-ligand 1 (PD-L1) inhibitor in patients with pMMR EC is less clear and may provide an opportunity for the addition of PARPi.^52^

PARPi combined with immunotherapy in EC has long been a therapeutic strategy of interest. The main rationale being that tumors exhibiting HRD have an increased tumor mutational burden leading to higher neo-antigen levels, which increase the anti-tumor immune response.^53^ Second, PARP inhibition upregulates PD-L1 expression, and in the absence of a functional BRCA pathway there is activation of the innate immune response *via* the STING/gas pathway.^54^ The role of the addition of PARPi to immunotherapy was investigated in the DUO-E and RUBY part II studies.

### Clinical trials incorporating PARPi in EC

#### First line and maintenance treatment

DUO-E^55^ was a randomized placebo controlled phase III trial in patients with metastatic or recurrent EC evaluating the combination of the PD-L1 inhibitor durvalumab with carboplatin and paclitaxel followed by maintenance durvalumab alone or durvalumab in combination with Olaparib. Each experimental arm was compared to the control arm, carboplatin/paclitaxel. Median PFS was 9.6 months in the control arm, 10.2 months in the durvalumab arm, and 15.1 months in the durvalumab/olaparib arm. Statistically significant improvement for both experimental arms compared to chemotherapy alone was demonstrated: durvalumab HR 0.71; 95% CI, 0.57–0.89; *p* = 0.003 and olaparib/durvalumab HR 0.55; 95% CI, 0.43–0.69; *p* < 0.0001.^55^ RUBY part 2 (reported in abstract only) added niraparib to dostarlimab maintenance previously investigated in RUBY part 1 with a comparison to carboplatin/paclitaxel/placebo. Similar to DUO E, a PFS benefit was seen for niraparib/dostarlimab over chemotherapy alone HR 0.60; 95% CI, 0.43–0.82; *p* = 0.007 in the overall patient population.^56^ Neither study was designed to provide a comparison between PARPi/immunotherapy and immunotherapy alone. In addition, neither study included a PARPi alone arm for comparison. These limitations in study design create a challenge when interpreting the data for the clinic.

In both studies pre-specified subgroup analyses, the dMMR population demonstrated benefit compared to chemotherapy alone. However, given the PFS benefit of this population with immunotherapy alone in both DUO E (HR 0.42 with durvalumab alone^57^) and RUBY part 1 (HR 0.28 with dostarlimab alone^51^), even without a direct statistical comparison, it does not appear that the dMMR patient population will derive additional benefit from a PARPi. What remains to be seen is whether a PARPi combination might be a successful approach for the approximately 30% of dMMR patients who progress on immunotherapy or, as part of a second line strategy, when patients progress following immunotherapy maintenance.

In both DUO E and RUBY part 2, the combination of PARPi and immunotherapy in the pMMR subgroup demonstrated benefit compared to control: Olaparib/durvalumab HR 0.57, 95% CI, 0.44–0.73; no *p*-value supplied, and niraparib/dostarlimab HR 0.63, 95% CI, 0.44–0.91, *p* = 0.006.^56^ These were planned subgroup analyses; however, the pMMR subgroup is heterogenous, and include NSMP and P53abn molecular subgroups which, together with the study designs, make interpretation challenging. Further exploratory analyses from both studies suggest that there may be patient populations that derive benefit from the addition of a PARPi. In DUO E, patients with ‘potentially pathogenic’ mutations in HRR genes (FoundationOne CdX, Cambridge, Massachusetts) had an improvement in PFS with the addition of olaparib HR 0.30, 95% CI, 0.15–0.58 (no *p*-value supplied).^57^ In RUBY part 2, patients with P53abn EC appeared to be doing better HR 0.29, 95% CI, 0.13–0.63 (no *p*-value supplied). These data are hypothesis generating. Further work is necessary to understand if there are subgroups of patients that derive benefit from PARPi maintenance and whether they require PARPi alone or in combination with immunotherapy. Future studies should be used to define and validate an appropriate HRD assay for use in EC.

The PARPi were generally well tolerated in both studies with no unexpected toxicities with the exception of pneumonitis which occurred in 12 (5%) patients in the DUO E study receiving olaparib and durvalumab.^57^ This was not seen in the comparable ovarian study and warrants further follow-up in subsequent immunotherapy/PARPi trials. Secondary MDS/AML has not been reported in either trial, however, longer follow-up is required to fully assess the risk in the EC patient population.

The UTOLA trial,^55^ published in abstract only, investigated the role of maintenance PARPi in EC. In this randomized phase IIb trial, 147 patients who had not progressed on platinum/paclitaxel chemotherapy for recurrent or metastatic disease were randomized to either olaparib or placebo. Treatment was continued until PD or intolerance. The median PFS was 5.6 months (95% CI, 3.8–7.4) in the olaparib arm and 4.0 months (95% CI, 3.6–7.4) in the placebo arm HR 0.94, *p* = 0.29. Tumors exhibiting HRD were found in 52% of patients, as defined by an assay that measured chromosome instability by large-scale genomic events (LGEs). A score of ⩾6 was the cut off assigned by the investigators, and we await a fuller description of this assay in the manuscript. In a pre-specified analysis in patients whose EC had a LGE score ⩾6 (*N* = 73), patients receiving olaparib experienced a significantly longer median PFS of 5.36 months (95% CI, 3.6–9.6) compared to 3.6 months (90%, 1.8–4.9) with placebo, HR 0.59, 95% CI, 0.41–0.71, *p* = 0.021. No significant difference in overall survival (OS) was observed across all subgroups. In the HR ‘proficient’ EC population, no benefit was seen from olaparib HR 1.27, 95% CI, 0.73–2.21, *p* = 0.193. At this time, the role for single-agent PARPi as a maintenance strategy in EC has not been established.^55^

#### Pre-treated recurrent metastatic EC

PARPi have been investigated in a number of phase I and II studies in unselected, previously treated, metastatic EC patient populations, summarized in Table 1. To date, single-agent PARPi in this EC patient population appears to have very limited activity. Single-agent niraparib resulted in an overall response rate (ORR), *n* = 25, of 4% (95% CI, 0–20) with a clinical benefit rate of 20%. No significant associations were detected between clinical benefit and IHC markers (PTEN, P53, MMR, PD-L1) or molecular profiling (*PTEN*, *P53*, HRR genes).^58^ The response rate and PFS in this study were similar to that seen in the olaparib only arm of NRG GY012, with a median PFS of 2 months (95% CI, 1.8–4.7) compared to 3.8 months (95% CI, 3–5.4) with the reference arm cediranib HR 1.45, 95% CI, 0.43–1.14.^39^

**Table 1.** Clinical trials regarding the use of PARPis in EC.

Author	Phase	Combination	Condition (*n* = EC/total)	Inclusion/biomarkers for inclusion	Main results regarding EC
Romero *et al.* (2020)^59^	WoO	Neoadjuvant Olaparib WoO trial to assess translational effects of Olaparib 300 mg BID for 28 days before surgery on early EC.	EC (*n* = 31)	Early EC/all comers.	*In vivo* evidence that olaparib may be more effective in ARID1A mutated patients.
Zimmer *et al.* (2019)^60^	I	Olaparib + Durvalumab + Cediranib RP2D was Cediranib 20 mg (5 days on/2 days off) + Durvalumab 1500 mg 14w + Olaparib 300 mg BID.	Solid tumors including EC (*n* = 1/9)	Recurrent/all comers.	PR for 7 months for the EC patient.
Poveda *et al.* (2021)^61^	I	Olaparib + Lurbinectedin RP2D was lurbinectedin 1.5 mg/m^2^ + olaparib 250 mg BID.	Solid tumors including EC (*n* = 5/20)	Recurrent/all comers.	SD 60%, no CR or PR.
You *et al.* (2022)^62^	I–II	Olaparib + Cyclophosphamide + Metformin Metronomic cyclophosphamide 50 mg PO QD and metformin 500 mg PO TID. RP2D was Olaparib 300 mg BID	EC (*n* = 31, but included 2 carcinosarcomas)	Recurrent/all comers	ORR 20.8%; PFS 5.1 m; Disease control rate 66.6%.
Westin *et al.* (2021)^63^	Ib	Olaparib + Capivasertib DL1 was the same as RP2D due to diarrhea and vomiting: Olaparib 300 mg PO BID and Capivasertib PO BID on a 4-day on 3-day off schedule.	Solid tumors including EC (*n* = 11/38)	Recurrent/all comers.	ORR for EC 44% (4/9) one with SD for more than 4 months.
Yap *et al.* (2022)^64^	Ib	Rucaparib + Sacituzumab-govitecan Cohort 1 Rucaparib 300 mg PO SID and cohort 2 Rucaparib 300 mg PO BID.	Six patients, 1 EC *BRCA*-mutated	Recurrent/all comers.	The combination was well tolerated. EC had a PR for 24 weeks.
Post *et al.* (2022)^65^	II	Olaparib + Durvalumab Single-arm Durvalumab 1500 mg IV q4w and Olaparib 300 mg BID.	EC (*n* = 55)	Recurrent/all comers.	PFS6 34%; PFS 3.4 months; OS 8.0 months; ORR 16%; in total 1CR and 7PR.
Konstantinopoulos *et al.* (2022)^66^	II	Talazoparib + Avelumab Single-arm Talazoparib 1 mg PO SID + Avelumab 10 mg/kg IV q2w.	EC (*n* = 35)	Recurrent/pMMR.	CBR 25%; ORR 11.4%; PFS6 22.9% HRD was related to clinical benefit.
Jackson *et al.* (2022)^67^	II	Rucaparib + Bevacizumab Single-arm Rucaparib 600 mg PO BID + Bevacizumab 15 mg/kg on D1 q21d.	CC (*n* = 5) and EC (*n* = 23)	Recurrent/all comers.	PFS6 30%; ORR 14% ARID1A mutated had better outcomes PTEN mutated had higher ORR but shorter PFS6.
Thavaneswaran *et al.* (2023)^68^	II	Olaparib + Durvalumab Single-arm Olaparib 300 mg PO BID + Durvalumab 1500 mg IV q 28d.	Solid tumors including EC (*n* = 2/48)	Recurrent/HRD mutated.	One CR in an EC patient with CHECK2.
Madariaga *et al*. (2023)^58^	II	Niraparib ± Dorstalimab Open label 2 cohort trial with either Niraparib 200 or 300 mg PO SID (based on baseline body weight and platelet count) (*n* = 25) or niraparib (same dose and schedule) with Dostarlimab 500 mg IV q3w for four cycles, followed by 1000 mg q6w thereafter (*n* = 22).	EC (*n* = 47)	Recurrent/all comers.	Niraparib alone: CBR 2% and ORR 20% Niraparib combination: CBR 31.8% and ORR 14%. No significant associations with IHC markers or molecular profiles.
Rimel *et al.* (2023)^39^ (NRG-GY12 trial)	II	Olaparib + Cediranib Randomized 1:1:1 phase II trial to Cediranib 30 mg SID or Olaparib 300 mg BID or Cediranib 30 mg SID with Olaparib 200 mg BID.	EC (*n* = 120)	Recurrent/all comers.	mPFS for cediranib was 3.8 months compared to 2.0 months for olaparib [HR 1.45 (95% CI, 0.91–2.3) *p* = 0.935], and 5.5 months for olaparib/cediranib (HR 0.7, 95% CI, 0.43–1.14; *p* = 0.064). Four patients receiving the combination had a durable response lasting more than 20 months.
Joly Lobbedez *et al.*, (2023)^55^ (UTOLA trial)	IIb	Olaparib maintenance Randomized phase II to maintenance Olaparib 300 mg BID or placebo for patients who achieved disease control after chemotherapy until disease control.	EC (*n* = 147)	Advanced or metastatic/all comers.	mPFS in the ITT and olaparib was 5.6 and 4.0 months, respectively (HR 0.94, *p* = 0.29). No difference in OS.
Westin *et al.* (2023)^57^ (DUO-E trial)	III	Olaparib + Durvalumab maintenance Carboplatin/paclitaxel/durvalumab placebo IV q3w for six cycles, followed by maintenance durvalumab placebo plus olaparib placebo (control arm) or carboplatin/paclitaxel/durvalumab 1120 mg IV q3w for six cycles, followed by maintenance durvalumab 1500 mg IV q4w plus olaparib placebo BID (durvalumab arm); or platinum-based chemotherapy plus durvalumab 1120 mg IV q3w for six cycles, followed by maintenance durvalumab 1500 mg IV q4w plus olaparib 300 mg BID (durvalumab + olaparib arm).	EC (*n* = 718)	Newly diagnosed advanced or newly recurrent after 12 months of adjuvant chemotherapy/all comers.	mPFS for control, durvalumab, and durvalumab + olaparib arms in the ITT was, respectively, 9.6, 10.2, and 15.1 months. HR significant for both durvalumab (HR 0.71; 95% CI, 0.57–0.89; *p* = 0.003) and the combination of durvalumab and olaparib (HR 0.55; 95% CI, 0.43–0.69; *p* < 0.0001). OS is not mature, but tendency to be positive.
Mirza *et al.* (2024)^56^ (RUBY Part 2 trial)	III	Niraparib + Dostarlimab maintenance Carboplatin/paclitaxel/dostarlimab placebo IV q3w for six cycles, followed by maintenance dostarlimab placebo + niraparib placebo (control arm) or carboplatin/paclitaxel/dostarlimab 500 mg IV q3w for six cycles, followed by maintenance dostarlimab 1000 mg q6w up to 3 years + niraparib 200 or 300 mg daily up to 3 years.	EC (*n* = 291)	Newly diagnosed advanced or newly recurrent after 6 months of adjuvant therapy/all comers.	PFS12 in the overall population 33.7% *versus* 57% (HR 0.60; 95% CI, 0.43–0.82; *p* = 0.007). PFS12 in the pMMR population 31.1% *versus* 54.7% (HR 0.63; 95% CI, 0.44–0.91; *p* = 0.006). PFS12 in the dMMR population 40.8% *versus* 64.4% (HR 0.48; 95% CI, 0.24–0.96; *p* = 0.0174).

BID, twice a day; CBR, clinical benefit rate; CC, cervical cancer; CI, confidence interval; CR, complete response; dMMR, mismatch repair deficient; EC, endometrial cancer; HRD, homologous recombinant deficiency; HR, hazard ratio; IHC, immunochemistry; IIT, intention to treat; IV, intravenous; ORR, overall response rate; OS, overall survival; PARP, poly(ADP-ribose) polymerase; PARPi, PARP inhibitor; PFS, progression-free survival; PFS6, progression-free survival at 6 months; PFS12, progression-free survival at 12 months; pMMR, MMR proficient; PO, oral; PR, partial response; QD, once a day; q2w, every two weeks; q6w, every 6 weeks, QID, four times a day; RP2D, recommended phase II dose; SD, stable disease; SID, once daily; TID, three times a day; WoO, window of opportunity.

The combination of immunotherapy with PARPi in a recurrent pre-treated patient population demonstrated modest efficacy. Konstantinopoulos *et al*.^66^ explored the combination of talazoparib and the PD-L1 inhibitor avelumab in patients with recurrent pMMR EC (Table 1). Of the enrolled patients, 25.7% derived clinical benefit, with an ORR of 11.4%, and a 22.9% PFS rate at 6 months. HR status, defined by known pathogenic mutations in HRR genes, suggested that these patients had a better outcome *p* = 0.01. In this study, six patients had potentially pathogenic somatic mutations: *CDK12* (*n* = 2); *BRCA1*, *BRCA2*, *BRIP1*, and *FANCA*.^66^ Additional PARPi combination studies with immunotherapy are described in Table 1. Further exploration of immunotherapy/PARPi combinations (including with anti-angiogenic agents), regimen sequencing, and identification of predictive biomarkers are warranted to fully understand the scope this therapeutic strategy in EC.

The combination of PARPi and agents targeting angiogenesis has been extensively investigated in ovarian cancer. Anti-angiogenic agents downregulate genes involved in hypoxic stress in pre-clinical models, potentially leading to synergy with PARPi.^69^ NRG-GY012 evaluated the combination of olaparib and the RTKi cediranib compared to the reference arm cediranib in a pre-treated EC patient population. A *post hoc* exploratory analysis of HR status evaluated HRR genes (BROCA-GO panel) and gLOH with a cut off >11%. This cutoff was determined specifically for this assay based on testing ovarian cancer samples with known HRD status. The combination of olaparib/cediranib resulted in a median PFS of 5.5 *versus* 3.8 months for cediranib, which did not meet statistical significance. Nonetheless, four patients (10%) receiving olaparib/cediranib did experience a durable response lasting more than 20 months. This study highlighted the challenges of using archival formalin-fixed paraffin-embedded samples, as the number of samples suitable for analysis were limited. As previously discussed,^70^ no association with outcome and HRR gene mutation or gLOH (BROCA-GO assay) were observed.^39^

The combination of rucaparib and the monoclonal antibody targeting vascular endothelial growth factor, bevacizumab, was investigated in a single-arm phase II trial including pretreated EC (*N* = 23) and cervical cancer (CC) (*N* = 5) patients. In the EC cohort, the six month PFS was 30% (95% CI, 0.1–0.5) with an ORR of 14%. FoundationOne NGS identified *ARID1A* mutations in five EC patients. In these five patients, the ORR was 33% and 6-month PFS 66.7% (Table 1).^67^ Further studies are exploring the impact of *ARID1A* mutations in PARPi combinations, niraparib and bevacizumab (NCT05523440) and ceralasertib with olaparib (NCT03682289) (Table 2).

**Table 2.** Trials registered on Clinicaltrials.gov in October 2023 regarding EC and PARPis.

Title (NCT number)	Phase	Interventions	Condition	Biomarkers
Testing the combination of DS-8201a and Olaparib in HER2-expressing cancers with expansion in patients with EC (NCT04585958)	I	DS-8201a and Olaparib	EC	HER2 over-expressed
Niraparib and Copanlisib in treating patients with recurrent endometrial, ovarian, primary peritoneal, or fallopian tube cancer (NCT03586661)	I	Best dose and side effects of Niraparib and Copanlisib	EC Ovarian Fallopian Peritoneal	BRCA mutated
Olaparib in combination with carboplatin for refractory or recurrent women’s cancers (NCT01237067)	I	To determine the safety and effectiveness of combined carboplatin and Olaparib as a treatment for gynecologic or breast cancer	EC Ovarian Breast Peritoneal Fallopian	All comers
Effects of PARP inhibitor on tumor microenvironment in high-risk EC patients (NCT05320757)	I	Window of opportunity for treatment-naïve patients to receive Olaparib before definitive treatment. The aim is to evaluate the DNA damage and inflammatory response after PARPi.	EC	All comers
A study of targeted agents for patients with recurrent or persistent EC (EndoMAP) (NCT04486352)	I–II	Atezolizumab + a second drug depending on the molecular profile. One cohort with Talazoparib	EC	All comers
Study of AZD5305 as monotherapy and in combination with anti-cancer agents in patients with advanced solid malignancies (NCT04644068)	I/IIa	Experimental ARP inhibitor AZD5305 alone or in combination	Solid tumors	All comers
Study of CYH33 in combination with Olaparib an oral PARP inhibitor in patients with advanced solid tumors (NCT04586335)	Ib	CYH33, an oral PI3K inhibitor, in combination with Olaparib	Solid tumors	Any DDR gene or PIK3CA mutation
mTORC1/2 inhibitor AZD2014 or the oral AKT inhibitor AZD5363 for recurrent endometrial and ovarian (NCT02208)	Ib/II	Side effects and best dose of Olaparib and Vistusertib (AZD2014) or Olaparib and Capivasertib (AZD5363) when given together	Solid tumors	All comers
Study evaluating the efficacy of a double immunotherapy combined with olaparib in patients with solid cancers and carriers of homologous recombination repair genes after Olaparib treatment (GUIDE2REPAIR) (NCT04169841)	II	Olaparib (300 mg BID) for 6 weeks, in the absence of PD after this time they had Olaparib and immunotherapy by Durvalumab (1500 mg Q4W) + Tremelimumab (75 mg IV Q4W) for 4 months, followed by Durvalumab alone until disease progression, death, intolerable toxicity, or patient/investigator decision to stop (for a maximum duration of 24 months).	Solid tumor including EC	HRD mutated
A study of Pembrolizumab and Olaparib in people with EC or endometrial carcinosarcoma (NCT05156268)	II	Olaparib + Pembrolizumab in advanced EC or endometrial carcinosarcoma	EC Carcinosarcoma	All comers
Trial of maintenance with niraparib – uterine serous carcinoma (NCT04080284)	II	Niraparib as maintenance for serous EC	EC	All comers
Bevacizumab and/or Niraparib in patients with recurrent endometrial and/ or ovarian cancer with ARID1A mutation (ARID1A) (NCT05523440)	II	Niraparib + Bevacizumab	EC or ovarian with ARID1A mutation	ARID1A
Testing the use of the combination of Selumetinib and Olaparib or Selumetinib alone targeted treatment for RAS pathway mutant recurrent or persistent ovarian and ECs, a ComboMATCH treatment trial (NCT05554328)	II	Selumetinib plus Olaparib compared to Selumetinib alone	EC Fallopian Ovarian Peritoneal	RAS mutated
Ceralasertib (AZD6738) alone and in combination with Olaparib or Durvalumab in patients with solid tumors (NCT03682289)	II	Ceralasertib alone or in combination with Olaparib or Durvalumab	Selected solid tumors including EC	ATM cohort ARID1A cohort
Rucaparib *versus* placebo maintenance therapy in metastatic and recurrent EC (NCT03617679)	II	Rucaparib as maintenance after 1 or 2 lines of therapy	EC	All comers
Does Cediranib with paclitaxel, or Cediranib and Olaparib, treat advanced EC better than paclitaxel? (COPELIA) (NCT03570437)	II	Paclitaxel ± Cediranib ± Olaparib	EC Carcinosarcoma	All comers
Avelumab in patients with MSS, MSI-H, and POLE-mutated recurrent or persistent EC and of Avelumab/Talazoparib and Avelumab/Axitinib in patients with MSS recurrent or persistent EC (NCT02912572)	II	Avelumab alone and in combination with Talazoparib or Axitinib in metastatic EC	EC	All comers
Study of Niraparib and TSR-042 in recurrent EC (NCT03016338)	II	Anti-PD1 inhibitor TSR-042 + Niraparib	EC	All comers
Testing the addition of an immunotherapy drug, tremelimumab, to the PARP inhibition drug, olaparib, for recurrent ovarian, fallopian tube or peritoneal cancer (NCT04034927)	II	Olaparib with or without tremelimumab	Ovarian Fallopian Peritoneal OR all if BRCA mutated	All comers for ovarian, fallopian and peritoneal. If BRCA mutated, all histologies
Combination Niraparib and Dostarlimab therapy for recurrent or persistent uterine serous carcinoma (NCT05870761)	II	Niraparib and Dorstalimab	EC Adenocarcinoma	All comers
Testing the combination of Olaparib and Durvalumab, Cediranib and Durvalumab, Olaparib and Capivasertib, and Cediranib alone in recurrent or refractory EC following the earlier phase of the study that tested Olaparib and Cediranib in comparison to cediranib alone, and Olaparib alone (NCT03660826)	II	Combination of Olaparib and Durvalumab, Cediranib and Durvalumab, Olaparib and Capivasertib, and Cediranib alone	EC	All comers
The EndoBARR trial (Endometrial Bevacizumab, Atezolizumab, Rucaparib) (EndoBARR) (NCT03694262)	II	Efficacy and safety of the combination of Rucaparib, Bevacizumab, and Atezolizumab in recurrent EC	EC and uterine carcinosarcoma	All comers
Refining adjuvant treatment IN EC based on molecular features (RAINBO) (NCT05255653)	II–III	Umbrella program with one arm for p53 abnormal EC	EC	p53 abnormal
Recurrent ovarian CarcinoSarcoma anti-pd-1 Niraparib (NCT03651206)	II/III	TSR-042 (anti-PD-1 mAb) in combination with Niraparib *versus* Niraparib alone compared to chemotherapy	EC and ovarian carcinosarcoma	All comers
A study of various treatments in serous or p53 abnormal endometrial cancer (CAN-STAMP) (NCT04159155)	II/III	For advanced EC, niraparib will be given as maintenance *versus* observation	EC	P53 abnormal
Durvalumab with or without olaparib as maintenance therapy after first-line treatment of advanced and recurrent EC (DUO-E) (NCT04269200)	III	Durvalumab with or without Olaparib as maintenance after first-line chemotherapy	EC	All comers

BID, twice a day; BRCA, BReast CAncer gene; DDR, DNA damage response; EC, endometrial cancer; HER2, human epidermal growth factor receptor 2; EC, endometrial cancer; HRD, homologous recombinant deficiency; IV, intravenous; OR, odds ratio; PARP, poly(ADP-ribose) polymerase; PARPi, PARP inhibitor; PD, progression of disease; PI3KCA, phosphoinositide 3-kinase; RAS, Rat sarcoma.

Mutations in the phosphoinositide 3-kinase (PI3K)/Akt/mammalian target of rapamycin (mTOR) pathway are frequent across EC molecular subgroups.^71^ In pre-clinical models, treatment with a PARPi resulted in increased expression of PI3K/Akt/mTOR pathway members across various tumor models.^72,73^ Furthermore, blocking the PI3K/Akt/mTOR pathway resulted in reduced HRR by suppressing BRCA1/2 expression enhancing sensitivity to PARPi.^74^ These pre-clinical data led to a multi-disease site clinical trial^64^ combining olaparib with the AKT inhibitor capivasertib. Eleven patients were included with EC, the ORR was 44% (4/9), with one patient achieving stable disease (SD) for more than 4 months (Table 1).^63^ This combination is currently being further evaluated in the second phase of the NRG-GY012 trial (NCT03660826).

#### Early phase trials and translational studies

EC patients have been included in several phase I studies exploring PARPi combinations (Table 1). Zimmer *et al.*^60^ conducted a phase I study of durvalumab, olaparib, and cediranib. One patient with pMMR EC was included experiencing a partial response (PR) for 7 months. In this study, while tumoral PD-L1 expression correlated with clinical benefit, no notable effects on cytokines or peripheral immune subsets were observed.^60^

The combination of rucaparib with the antibody drug conjugate (ADC) sacituzumab-govitecan was investigated in a small cohort of six patients, one of whom had EC. This patient had a pathogenic *BRCA1* mutation and had previously received niraparib maintenance post-chemotherapy. The patient experienced a sustained PR for 24 weeks (Table 1).^64^ Given the increasing interest in ADC as a treatment for all gynecologic cancers, understanding whether there is a rationale for combining PARPi with these agents is of interest.

Window of opportunity (WoO) studies provide an opportunity to understand the biology associated with drug delivery and can provide valuable information to direct combination studies. In 2020, Romero *et al*.^59^ conducted a WoO study including 31 EC patients who received olaparib for 28 days prior to surgery. The primary endpoints were translational, measuring changes in cell cycle-related proteins cyclin D1, Ki67, and cleaved caspase-3 between biopsy and surgical specimen. Significant inhibition of cyclin D1 (*p* < 0.01) was observed, with no significant changes in Ki67 and active caspase 3 immunostaining. PARP-1 levels correlated positively with cyclin D1 levels (rho = 0.661, *p* = 0.0001). Both PARP-1 and cyclin D1 levels were significantly lower in *ARID1A* negative tumors compared to *ARID1A* positive tumors (*p* = 0.022 and *p* = 0.004, respectively). Overall, the study suggests that olaparib may be more likely to be effective in *ARID1A* mutated tumors. No information regarding HRD or BRCA status was disclosed (Table 1).^59^ As described previously, other studies are ongoing in this patient population (Table 2).

### EC and PARPi future directions

Understanding if there is an HRD phenotype and biomarker for PARPi activity in EC will require ongoing pre-clinical as well as translation research. In October 2023, there were 12 trials including PARPi actively recruiting EC patients (Table 2). Notably, most of these trials explore PARPi in combination with other targeted agents. Many of these studies pre-select patients on the basis of biomarkers associated with the companion agent, for example, HER2 overexpression for the combination of olaparib with Trastuzumab-deruxtecan (NCT04585958). It will be important to understand from the translational work associated with these trials if there are biomarker signatures that inform the use of PARPi.

Strong pre-clinical data exist suggesting that PARPi may be an effective radiosensitizer given its inhibition of DNA repair, inhibition of chromatin remodeling, putative vasodilatory effect (impacts hypoxia), and G2-M arrest cooperation.^75^ The role of PARPi as a radiosensitizer in EC and in CC is currently being evaluated (NCT03968406).

Finally, extrapolating from ovarian cancer, PARPi maintenance may hold promise in the frontline/adjuvant setting. Although whether this will be alone or in combination with immunotherapy is unclear. The molecular subgroup-specific RAINBO set of adjuvant studies (NCT05255653) incorporates PARPi maintenance in the P53abn-RED trial investigating adjuvant chemoradiation with olaparib *versus* chemoradiation alone (Table 2). A similar approach is being taken with niraparib in the CAN STAMP study (NCT 04159155). These academic studies incorporate extensive tissue collections which will enable translational research and discovery.

## Uterine leiomyosarcomas

Uterine leiomyosarcomas (uLMS) account for approximately 3–7% of all uterine cancers. They are characterized by aggressive biological behavior, resulting in early local and distant metastatic dissemination. Although surgery can be curative for approximately 50% of early-stage cases, advanced or recurrent disease shows minimal responsiveness to current standard treatments. To date, adjuvant strategies have not proven to be beneficial.^76^

A comprehensive analysis of 83 uLMS samples including whole-exome sequencing (WES), RNA-sequencing (RNA-Seq), and whole-genome sequencing (WGS) found recurrent somatic mutations in *P53*, *MED12*, and *PTEN* genes. Somatic copy number variation analysis identified notable gains and losses, including amplifications in *TERT*, *C-MYC*, and *MYOCD/MAP2K4*, as well as various copy-number losses. An analysis using SigProfiler on 48 fresh frozen tumor-normal pairs identified the HRD SBS3 signature in 25% of uLMS tumors. This specific mutational signature, known for its correlation with *BRCA1* and *BRCA2* biallelic inactivation and HRD in various solid cancers, was detected in 12 tumors. Only four of these tumors harbored either germline or somatic mutations in HRR genes, suggesting that a potential epigenetic mechanism of loss may be occurring in uLMS.^77^ Pre-clinical uLMS (exhibiting HRD) patient derived xenograft models demonstrated rapid and sustained response to PARPi. AZD5305 was more effective than either olaparib or olaparib with cisplatin. This was observed even in a BRCA2-deleted patient-derived xenograft (PDX) model derived from a patient’s tumor following PARPi treatment. This pre-clinical work illustrates the importance of PDX in the context of rare malignancies. Although further work is required to fully optimize identification of an HRD phenotype in uLMS, these data provided support for clinical trials with PARPis for patients with uLMS.^78^

NCI 10250 is a phase II trial that assessed the combination of trabectedin and olaparib in advanced uLMS in 22 pre-treated patients. All patients underwent biopsy, had WES, and a functional RAD51 foci assay to evaluate for HR status. Notably, 59% of the patients had received ⩾3 prior lines of treatment. The ORR was 27% (6 of 22) with a median PFS of 6.9 months (95% CI, 5.4 months to not estimable). A pathogenic HRR gene mutation was observed in 31% of tumors, and 50% demonstrated HRD on the basis of the RAD51 foci assay. Toxicity was manageable with dose modification. In an exploratory analysis, patients with HRD based on RAD51 foci had prolonged PFS compared to those with HR proficient tumors, 11.2 *versus* 5.4 months, *p* = 0.05.^79^ Furthermore, two case reports suggest a benefit for PARPi in BRCA mutated uLMS.^80,81^

A phase II/III trial for patients with pre-treated uLMS is currently recruiting patients. It aims to compare the efficacy of the combination of olaparib and temozolomide with investigator’s choice of trabectedin or pazopanib (NCT03880019).

Better understanding of HRD in the context of the broader uLMS patient population is required together with validation of a suitable signature for clinical use.

## Cervical cancer

CC continues to be a global health problem with an estimated 603,863 new cases diagnosed each year.^29^ There are two main histological subtypes, squamous cell carcinoma (SCC), accounting for 80% of cases, and adenocarcinoma. Persistent infection with human papilloma virus (HPV) is still the primary factor leading to CC, irrespective of histological subtype.^82,83^

Initial management of CC is surgery (stage 1A/B1) or for later stage disease the combination of chemo- and radiotherapy. Exploration of strategies including PARPi to improve the efficacy of frontline treatment either in maintenance or as a radiosensitizer are of interest. For advanced, recurrent, or metastatic disease the current standard of care includes carboplatin, paclitaxel, and bevacizumab, with or without pembrolizumab based on PD-L1 positivity.^84^ Subsequent lines of treatment vary based on jurisdiction. However, options remain limited and enrollment in clinical trials is preferred.^85,86^ Notably, there are currently no established recommendations for the use of PARPi in the treatment of CC.

### The rationale for PARPi use in CC

**Figure 2.**
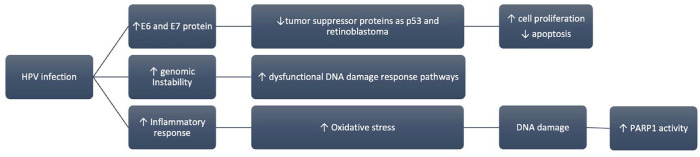
The role of HPV infection in the pathogenesis of CC. HPV encoded E6 and E7 oncoproteins disrupt key tumor suppressor proteins like P53 and retinoblastoma, fostering cell proliferation and impeding apoptosis. This, coupled with genomic instability often tied to malfunctioning DNA damage response pathways, contributes to tumorigenesis. Notably, PARPis exhibit effectiveness in cancers with DNA damage response deficiencies, exemplified in BRCA-mutated gynecological cancers. A second hypothesis implicates chronic inflammation induced by HPV infection in CC pathogenesis. HPV-triggered inflammatory responses culminate in oxidative stress and DNA damage. This chronic inflammation also leads to E6 overexpression, elevating DNA damage levels, and consequently enhancing PARP-1 activity. Both mechanisms provide potential targets for PARPi. CC, cervical cancer; HPV, human papilloma virus; PARPi, poly(ADP-ribose) polymerase inhibitors.

HPV infection in CC development involves several mechanisms that provide a rationale for the clinical investigation of PARPi (Figure 2). The HPV-encoded E6 and E7 oncoproteins interfere with key tumor suppressor proteins, such as P53 and retinoblastoma, promoting cell proliferation and inhibiting apoptosis.^87,88^ Additionally, genomic instability, often linked to dysfunctional DNA damage response pathways, contributes to tumorigenesis.^89^ A second hypothesis suggests that chronic inflammation triggered by HPV infection ultimately lead to oxidative stress and DNA damage, with E6 overexpression leading to higher levels of PARP-1 activity.^90^ Both mechanisms create a potential target for PARPi (Figure 2). Furthermore, ARID1A mutation (discussed under EC) occurs in 17% of cervical adenocarcinoma, with loss of expression occurring in approximately 12% of cases. ARID1A mutation occurs in 7% of SCC (loss of expression in approximately 12%), providing a further potential rationale.^44^ Again, specific CC data are extremely limited. One study with 150 cervix samples found minimal PARP-1 expression in normal cells, but positivity in 86% of low-grade, 77.5% of high-grade lesions, and 94% of invasive SCC. The study also suggested there was a correlation between PARP positivity and HPV-positive high-grade intraepithelial cells.^91^

PARPi have been proposed as potential radio sensitizing agent in CC. Xue *et al.* investigated niraparib in nude mice bearing HeLa CC tumors. The combination of niraparib and radiotherapy significantly reduced tumor volume compared to monotherapy in untreated mice. The tumor growth delay ranged from 23.33 to 39 days with combination therapy (*p* < 0.05). Univariate analysis indicated a prolonged time for tumor growth when radiotherapy was followed by intragastric Niraparib rather than intraperitoneal niraparib (*p* = 0.003). The combination therapy reduced levels of PARP-1 precursor, PARP-1 splicer, PAR, and RAD51 protein. Conversely, it showed high expression of cleaved caspase 3 and low expression of Ki-67. Supporting further investigation of PARPi as a radio sensitizing agent in CC.^92^

### Clinical trials incorporating PARPi in CC

#### Recurrent and metastatic CC

Clinical data on PARPi for CC is limited. The first, conducted by Kunos *et al.*^93^ in 2015, involved a phase I–II trial assessing the combination of veliparib with topotecan in 27 patients with recurrent CC (Table 3). PR was observed in 7% of patients. Four individuals experienced PD more than 6 months after initiating investigational therapy. Patients with low PARP-1 by IHC exhibited a longer PFS (HR 0.25; *p* = 0.02) and improved survival (HR 0.12; *p* = 0.005). Common grade 3 or higher treatment-related AEs included anemia (59%), thrombocytopenia (44%), leukopenia (22%), and neutropenia (19%).^93^ Further investigation of the role of PARP1 as a biomarker in CC is warranted.

**Table 3.** Clinical trials regarding the use of PARPi in CC.

Author	Phase	Combination	Condition (*n* = CC/total)	Inclusion/biomarkers for inclusion	Main results regarding CC
Thaker *et al*, 2017^94^	I	Veliparib + Paclitaxel + Cisplatin Standard 3 + 3 phase I dose escalation with paclitaxel 175 mg/m^2^ on day 1, cisplatin 50 mg/m^2^ on day 2, and escalating doses of Veliparib ranging from 50 to 400 mg PO BID on days 1–7 on 21 days cycles until progression. MTD was not reached.	CC (*n* = 34)	Recurrent/all comers	ORR 34% for all doses and 60% for the 400 mg dose. mPFS 6.2 m and mOS 14.5 m
Kunos *et al*, 2015^93^	I–II	Veliparib + topotecan Veliparib 10 mg PO daily + Topotecan (0.6 mg/m^2^) IV on days 1–5 of each 21 days cycle until PD or toxicity.	CC (*n* = 27)	Recurrent/all comers	The combination was safe and well tolerated. Two (7%) patients with PR and 4 (14%) PD after 6 months.
Jackson *et al*, 2022^67^	II	Rucaparib + Bevacizumab Single-arm Rucaparib 600 mg PO BID + Bevacizumab 15 mg/kg on D1 q21d.	CC (*n* = 5) and EC (*n* = 23)	Recurrent/all comers	PFS6 22%; ORR 17% ARID1A mutated had better outcomes PTEN mutated had higher ORR but shorter PFS6

BID, twice a day; CC, cervical cancer; EC, endometrial cancer; IV, intravenous; mOS, median overall survival; mPFS, median progression-free survival; MTD, maximum tolerated dose; ORR, overall response rate; PARPi, poly(ADP-ribose) polymerase inhibitor; PD, progression of disease; PFS6, progression-free survival at 6 months; PO, oral; PR, partial response.

The combination of a PARPi with chemotherapy in CC has been investigated in a phase I study of paclitaxel, cisplatin, and veliparib. The maximum tolerated dose was not reached, and the ORR across all dose levels was 34% (*n* = 29). Median PFS was 6.2 months, and OS was 14.5 months (Table 3).^94^

Lastly, as previously discussed, five CC patients were included in the phase II study by Jackson *et al.*^67^ to assess the combination of rucaparib and bevacizumab. The ORR was modest 17%, especially given known response rates to bevacizumab alone of 11%.^67^ However, further investigation of PARPi combinations with anti-angiogenic agents may be of interest given the low numbers of studies on this topic to date.

### CC and PARPi future directions

Pre-clinical data are required to better understand the context of HRD in CC. Pre-clinical models evaluating potential PARPi combinations would be of interest. In October 2023, there were five trials actively recruiting patients. However, only two trials specifically target CC patients (Table 4). CC patients are included in studies targeting several cancer sites with the newer generation of PARPi AZD5305 (NCT04644068). We have previously discussed the tumor agnostic rationale for combinations of immunotherapy, anti-angiogenic agents, and PARPi. Given frontline treatment for CC incorporates chemotherapy with both a PD-1 inhibitor and bevacizumab, there may be a role for further exploration of PARPi in the maintenance setting or in combination on progression. CC patients are currently included in ongoing combination studies with immunotherapy, for example, niraparib plus dostarlimab (NCT04068753). Consideration for inclusion of CC patients in biomarker selected studies is of key importance given the limited number of trials open to CC patients. Based on loss of ARID1A expression, both adenocarcinoma and SCC patients were eligible for the ATARI trial. This trial investigates the role of ATR and PARP inhibition. Evaluation of PARPi as radiosensitizers (talazoparib to radiotherapy in gynecological cancers, including CC, NCT03968406) in the clinic or as maintenance in high-risk patients following treatment (potentially in combination with immunotherapy given the data arising from KEYNOTE 18A^95^) are future directions where PARPi may demonstrate benefit.

**Table 4.** Trials registered on Clinicaltrials.gov in October 2023 regarding PARPi in CC.

Title (NCT number)	Phase	Interventions	Condition	Biomarkers
Talazoparib and radiation therapy in treating patients with locally recurrent gynecologic cancers (NCT03968406)	I	Talazoparib + Radiotherapy in advanced tumors	Gynecologic	All comers
Selumetinib and Olaparib in solid tumors (NCT03162627)	I	Olaparib + Selumetinib	Solid tumors, including CC	All comers
Study of AZD5305 as monotherapy and in combination with anti-cancer agents in patients with advanced solid malignancies (PETRA) (NCT04644068)	I–II	Experimental PARPi AZD5305 alone or in combination	Selected solid cancer, including CC	All comers
Niraparib in combination with Dostarlimab in patients with recurrent or progressive cervix cancer (STAR) (NCT04068753)	II	Niraparib + Dorstalimab	CC	All comers
Pembrolizumab and Olaparib in cervical cancer patients (NCT04483544)	II	Pembrolizumab + Olaparib in advanced or recurrent CC after standard chemotherapy	CC	All comers
Niraparib in the treatment of patients with advanced PALB2 mutated tumors (PAVO) (NCT05169437)	II	Niraparib in advanced tumors	Solid tumors with PALB2 mutation	PALB2
Pembrolizumab plus Olaparib in patients with recurrent cervical cancer (NCT04641728)	II	Olaparib + Pembrolizumab	CC	All comers
Bevacizumab and Rucaparib in recurrent carcinoma of the cervix or endometrium (Clovis-001) (NCT03476798)	II	Rucaparib + Bevacizumab	CC Endometrium	All comers

CC, cervical cancer; PARPi, poly(ADP-ribose) polymerase inhibitor.

## Vulvar and vaginal cancer

Subtypes of vulvar and vaginal cancer also occur in association with HPV infection. As previously explored in this review, HPV infection and potentially p53 mutational state (vulvar) may render tumors susceptible to PARP inhibition. Currently, there are no available data on the use of these drugs for the treatment of either disease. Given both cancers are managed incorporating chemo and radio therapy, inclusion of PARPi as a radiosensitizer may be of interest. The only currently available trial incorporating PARPi enrolling patients with vaginal cancer focuses on investigating talazoparib in combination with radiotherapy for gynecological cancers (NCT03968406). Once again, greater understanding of the biology of these rarer cancers is essential. The pre-clinical work in uLMS suggests that this is possible. Development and use of PDX especially given these tumors are often amenable to biopsy would aid discovery. Inclusion of vulvar and vaginal cancer patients in tumor agnostic biomarker-driven studies is encouraged.

## Conclusion

Although established as part of routine clinical practice for the treatment of patients with ovarian cancer, it is clear that PARPi, as a therapeutic strategy alone and, perhaps more importantly, in combination warrants ongoing investigation in patients with non-ovarian gynecologic malignancies. Greater understanding of HRR and HRD in the context of each tumor type is required. This review highlights the challenges of optimizing the use of targeted agents, not just PARPi, in rare cancers. Thoughtful pre-clinical work to optimize PARPi approaches and combinations is essential. Defining the HRD phenotype and potential predictive biomarkers will require collaborative effort as it is clear ‘one size’ does not fit all.

Despite strong pre-clinical evidence in EC, it remains to be seen where PARPi are best positioned. It seems unlikely that single-agent PARPi will be a successful strategy for advanced/recurrent disease, except in a potentially yet-to-be-defined very narrow subgroup of clearly and contextually defined HRD cancers. Combination treatment with immunotherapy and other agents looks to hold more promise. Collaborative efforts in translational research utilizing blood and tissue samples collected as part of completed studies will help accelerate our understanding of PARPi in the clinic. Better understanding of PARPi and its impact when combined with radiotherapy is of interest particularly for tumors like CC and EC.

Finally, it is important that we consider how patient factors might impact efficacy and the toxicity of these agents. Broad inclusivity and increased diversity of participants both in our clinical and biomarker studies are essential if we are going to provide clinically meaningful guidance for all our patients.

## Declarations

**Ethics approval and consent to participate:** Not applicable.

**Consent for publication:** Not applicable.

**Availability of data and materials:** Not applicable.
